# A Novel Machine-Learning Framework Based on a Hierarchy of Dispute Models for the Identification of Fish Species Using Multi-Mode Spectroscopy

**DOI:** 10.3390/s23229062

**Published:** 2023-11-09

**Authors:** Mitchell Sueker, Amirreza Daghighi, Alireza Akhbardeh, Nicholas MacKinnon, Gregory Bearman, Insuck Baek, Chansong Hwang, Jianwei Qin, Amanda M. Tabb, Jiahleen B. Roungchun, Rosalee S. Hellberg, Fartash Vasefi, Moon Kim, Kouhyar Tavakolian, Hossein Kashani Zadeh

**Affiliations:** 1 Biomedical Engineering Program, University of North Dakota, Grand Forks, ND 58202, USA; 2 SafetySpect Inc., Grand Forks, ND 58202, USA; 3 USDA ARS Environmental Microbial and Food Safety Laboratory, Beltsville Agricultural Research Center, Beltsville, MD 20705, USA; 4 Food Science Program, Schmid College of Science and Technology, Chapman University, Orange, CA 92866, USA; 5 Department of Mechanical Engineering, University of North Dakota, Grand Forks, ND 58202, USA

**Keywords:** fish species identification, machine learning, multi-mode spectroscopy, dispute models, data fusion, classification, fish fraudulence, deep learning

## Abstract

Seafood mislabeling rates of approximately 20% have been reported globally. Traditional methods for fish species identification, such as DNA analysis and polymerase chain reaction (PCR), are expensive and time-consuming, and require skilled technicians and specialized equipment. The combination of spectroscopy and machine learning presents a promising approach to overcome these challenges. In our study, we took a comprehensive approach by considering a total of 43 different fish species and employing three modes of spectroscopy: fluorescence (Fluor), and reflectance in the visible near-infrared (VNIR) and short-wave near-infrared (SWIR). To achieve higher accuracies, we developed a novel machine-learning framework, where groups of similar fish types were identified and specialized classifiers were trained for each group. The incorporation of global (single artificial intelligence for all species) and dispute classification models created a hierarchical decision process, yielding higher performances. For Fluor, VNIR, and SWIR, accuracies increased from 80%, 75%, and 49% to 83%, 81%, and 58%, respectively. Furthermore, certain species witnessed remarkable performance enhancements of up to 40% in single-mode identification. The fusion of all three spectroscopic modes further boosted the performance of the best single mode, averaged over all species, by 9%. Fish species mislabeling not only poses health-related risks due to contaminants, toxins, and allergens that could be life-threatening, but also gives rise to economic and environmental hazards and loss of nutritional benefits. Our proposed method can detect fish fraud as a real-time alternative to DNA barcoding and other standard methods. The hierarchical system of dispute models proposed in this work is a novel machine-learning tool not limited to this application, and can improve accuracy in any classification problem which contains a large number of classes.

## 1. Introduction

**Figure 1:**
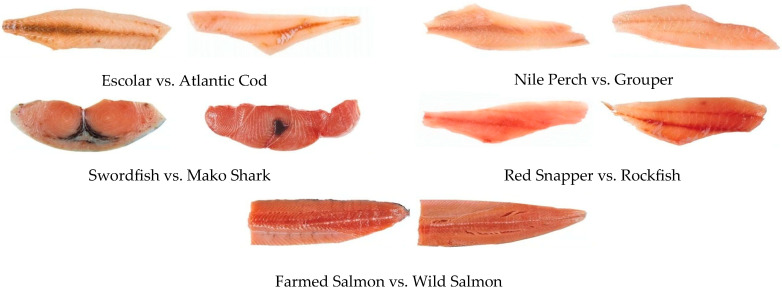
Many fish look alike when filleted (Adapted from [3]).

Seafood mislabeling has been reported in numerous studies globally. In a large-scale study, the nonprofit group Oceana found that 19% of fish sold in 55 different countries within all sectors of the supply chain were labeled as an incorrect species [1]. Fish mislabeling and fraud pose a grave threat to both consumers and the environment. The issue of illegal, unreported, and unregulated (IUU) fishing exacerbates this problem further, due to ineffective and inefficient fish assessment methods in the supply chain. Various deceptive practices are employed in fish fraud, including substituting expensive fish species with cheaper alternatives, falsely labeling frozen-thawed fish as fresh, misrepresenting the production method (such as claiming fish as wild-caught when it is actually farmed-raised), and falsifying the geographical origin of fish [2]. The risk of fish fraud is particularly high in fish fillets and portions, since they lack distinguishing features after processing, making it easy for fraudsters to deceive unsuspecting customers (Figure 1).

One of the most concerning aspects of fish fraud is the health risks it poses to consumers. All types of fish may pose dangers to consumers due to environmental contaminants, but species mislabeling can expose consumers to additional unexpected hazards. Unintentional consumption of allergens, toxins, contaminants, heavy metals, antibiotic residues, and other harmful substances due to mislabeling can result in severe food poisoning or life-threatening health issues, which can be misdiagnosed by end users due to the fraudulent species label (Table 1) [4]. Moreover, fish fraud has detrimental consequences for the environment. Mislabeling and IUU fishing can hinder conservation efforts and mislead management practices for specific fish populations, leading to overfishing of certain species and threatening marine ecosystems’ balance and biodiversity [5]. Economically, fish fraud creates imbalances in the seafood market. Consumers are charged premium prices for lower-quality fish, while legitimate fishers and seafood suppliers may suffer losses due to unfair competition. The fraudulent practice also undermines the efforts of honest businesses, discouraging consumers from trusting the authenticity of fish products and damaging the overall seafood industry’s reputation.

To combat this problem effectively, accurate fish assessment techniques and improved traceability are essential to ensure the sustainability and integrity of the seafood industry. Currently, the common techniques used for detecting fish species are visual inspection, DNA barcoding and real-time polymerase chain reaction (PCR). Visual inspection is not always reliable or accurate, and heavily depends on the level of expertise of the inspector, especially when the fish is stripped of external features. DNA barcoding which has been adopted by the U.S. Food and Drug Administration generally requires at least 1–2 days of laboratory work and analysis [6]. Real-time PCR is also popular, due to its portable and rapid evaluation, but it is a targeted approach that checks for a specific species, not a wide range of fish [7]. This makes its application limited when attempting to identify a large number of fish species. Table 2 compares current fish species identification methodologies. Each of these aforementioned approaches has its own disadvantages, such as being time-consuming, destructive to the fish, expensive, or requiring sample preparation and skilled operators. Due to these shortcomings, developing systems that can provide accurate and rapid on-site fraud detection is a necessity [8,9].

Spectroscopy offers portable and non-destructive solutions that do not need skilled workers. This method takes only seconds and can even measure through package plastic wrap. Spectroscopy has been a potential option for many years, but recent advancements in sensors coupled with machine learning algorithms and edge computing allow high accuracies and reliability when classifying the species of fish fillets and place it as a potential alternative to molecular approaches [9].

De Graeve et al. displayed a comprehensive dataset, proving spectroscopic systems can be very effective while being used in a real-world setting of monitoring a wide variety of fish [10]. Rapid Evaporative Ionization Mass Spectrometry (REIMS) was used to analyze 1736 samples containing 17 different fish species over seven years, and then 432 fish fillets were used to test the system with machine learning methods at the end of that period. An accuracy of 95% was achieved on the model containing all fish when classified using principal component analysis coupled with linear discriminant analysis (PCA-LDA). LDA was also employed by Qin et al., which, along with subspace discriminant analysis (SDA), obtained the highest performances in classifying six species at 100% accuracy [11]. The authors did this by acquiring line-scan hyperspectral images from fish fillets in four modes: reflectance in the visible and near-infrared region (VNIR), fluorescence (Fluor) by 365 nm UV excitation, reflectance in the short-wave infrared region (SWIR), and Raman by 785 nm laser excitation. Many different machine learning classifiers were utilized, as well as different types of datasets (full spectra, first ten components of principal component analysis, and bands selected by the sequential feature selection method), but the highest accuracies achieved for determining species was 100%, while utilizing the full VNIR reflectance spectra with LDA and SDA. Experiments conducted by Lv et al. further tested the efficacy of LDA when classifying fish species, while varying preprocessing methods to find the most successful approach [12]. Seven different freshwater species were examined, with 100 samples each, other than one species which contained 70 samples. Spectra to be analyzed were obtained through NIR spectroscopy, and classification accuracies of 100% were found while utilizing LDA models with multiple different preprocessing methods and data reduction techniques. Ren et al. proved the effectiveness of the fusion of multiple spectroscopic modes by combining laser-induced breakdown spectroscopy (LIBS) and Raman spectroscopy for fish species identification [13]. Thirteen different fish species were utilized, and support vector machine (SVM) and convolutional neural network (CNN) machine learning methods were used for classification. Low-, mid-, and high-level data fusion was used to combine the LIBS and Raman data. All three fusion strategies offered increases in accuracy, but the low-level fusion CNN model displayed the highest classification accuracy of 98.2%.

Three additional studies displayed how spectroscopic methods can be made more efficient for implementation within handheld systems. Handheld systems can improve the usefulness and provide applicability to the technology discussed earlier, as it offers a rapid and portable medium to classify fish species on-site. Chen et al. employed near-infrared spectroscopy (NIRS) to assess five different salmon and cod species, which numbered 500 in total [14]. A back propagation neural network (BNN) and a CNN were used for identifying fish species by the corresponding spectra obtained from NIRS. Different batch sizes, the number of convolutional kernels and layers, and the number of pooling layers on the spectra were compared. A 1D-CNN-8 model was deemed the most suitable for mixed fish, and reached accuracies of 98%. This algorithm is stated to be suitable for small mobile devices, and would allow for the implementation of edge computing for small NIR spectrometers. Chauvin et al. also utilized the VNIR region to obtain reflectance spectra, as well as to collect fluorescence spectra with UV illumination [15]. These data were analyzed from fourteen fish representing six species. Three to twenty-five selected wavelength bands were used for a spectral reconstruction algorithm, which allows for spectral imaging systems that will perform faster and are more cost-effective. Four different classifiers with five-fold cross-validation estimated the classification accuracy when determining the species. The spectral reconstruction algorithm with only three spectral bands garnered 99.98% and 99.94% with the linear discriminant and subspace discriminant classifiers, respectively. The same authors continued their work and expanded the dataset to 133 samples comprised of 25 different species [15]. Fluor, SWIR reflectance, and VNIR reflectance were used to capture narrow-band spectral data at three to seven wavelengths. Simulated annealing was applied to identify optimal wavelengths with a cost function based on the accuracy given from a weighted k-nearest neighbors (WKNN) classifier. A multi-layer perceptron (MLP) artificial neural network was trained on the data from each of the three different spectra, as well as the spectra from the different modes fused. The best accuracy obtained was 95% with fusion classification on seven wavelengths. Spectroscopy can also be used to assess fish freshness. Our team, Kashani Zadeh et al., obtained an accuracy of 95% using three modes of spectroscopy when classifying fish freshness within ±1 day from the catch date [16].

A challenge in machine learning for fish species identification and freshness assessment is that an increase in the number of classes makes achieving high accuracies difficult. Gupta et. al. showed that a high number of classes leads to class-confusability because they have more noise in their loss function, and proposed focusing on pairs of classes that are more easily distinguishable at any moment [17]. Thrampoulidis et. al. demonstrated that the accuracy of categorization relies significantly on the distribution, as various algorithms exhibit peak efficiency for distinct data distributions and/or dimensions of training features, and devised a theoretical method for overcoming those challenges [18]. Zhuravlev et. al. developed a two-tiered decision-making structure for multi-class recognition issues with large number of classes, founded on the enhancement of the error-correcting output codes (ECOC) technique [19]. They formed macro-classes by partitioning the original classes. This meant that for a new object, two stages of classification were performed. However, all of these studies only use the inputs as the segmentation criteria, and, to the knowledge of the authors, there are no studies that use the response for segmentation.

We utilized ensemble methods in our fish freshness study [16]. However, in fish species identification, the number of classes can be in the order of hundreds of species. Therefore, within this study, we are proposing and implementing a new multi-mode highly multi-class machine learning framework called system of dispute models. This technique trains a global model to identify groups of classes with feature subspaces too similar for a one-stage classification. Thus, we segment the overall space into smaller subspaces and train specialized models to better fit the dataset. A sample in the field will then be classified by the global model to identify the subspace, with the dispute model identifying the final class.

One way to understand the dispute model is to look at a similar problem using a standard color checker to either transform a red, green and blue (RGB) image into an International Commission on Illumination (CIE) color space or to obtain an International Color Consortium (ICC) color profile for a camera or printer to create standard color [20,21]. The standard color checker contains highly saturated blues, reds and yellows. These do not work well with a color object with a smaller gamut of colors (similar to the smaller spectral space of fish resolved with the dispute model). In the case of the color checker, creating an ICC profile or a CIE color space, the software attempts to optimize all colors, including those not in the sampled space. This creates larger errors across the board for all colors.

In our case, the artificial intelligence (AI) training includes fish that are not present in the reduced spectral space in the dispute model. By limiting the dispute models to only the species that challenge the global model, the dispute models operate in a reduced space differentiating between a few classes, rather than all classes. As an illustration of this in the color space example, there is a reduced-gamut color checker used for the cultural heritage image [22].

Our team is developing a handheld multi-mode point spectroscopy device for rapid, in situ, non-destructive, convenient, and accurate identification of fish species and freshness. This device, for the first time, will utilize the Fluor, VNIR, and SWIR spectroscopic modes. For proof of concept, we measured as many as 216 fish fillets from 43 different fish species using hyperspectral imaging in Fluor, VNIR and SWIR reflectance modes. We then developed an effective and efficient machine learning algorithm capable of differentiating different fish species by devising a novel machine learning technique that, after training and testing a general model, would group spectrally similar fish, and train specialized classifiers for each group. The single machine learning (ML) model that was trained on all fish types will be referenced as the global mode, and the models trained for each subgroup of spectrally similar fish are called dispute models. In the hybrid (global integrated with dispute) model, the global model called the appropriate dispute model in a hierarchical decision process. A patent application has been filed for this novel method [23].

## 2. Materials and Methods

### 2.1. Obtaining and Storing the Samples

The fish fillet samples used in this study were obtained from Fulton Fish Market [24], a reputable online seafood store, which allowed us traceability, repeatability, and accountability. A total of 43 different fish species, with a minimum of four sample fillets for each species, were selected, purchased and measured to provide a diverse representation of fish in the market. All acquired fish were in fillet or portion form. The selection of fillets instead of whole fish was due to the majority of all fish mislabeling occurring not on whole fish, but on fish in fillet and portion forms [25]. All fish were delivered frozen, and upon arrival placed in a −20 °C freezer. The samples were moved to a 4 °C refrigerator to thaw for 24 h prior to the imaging experiment. A piece of each sample was sent to Chapman University (Orange, CA, USA) for DNA barcoding [11] to identify the true species to be used as ground truth in our machine learning algorithm.

### 2.2. Data Acquisition Process

The data acquisition process in this study involved the collection of both fluorescence and reflectance spectra, in the VNIR and SWIR regions of 216 fish fillets, representing 43 different species groups. Table 3 shows the number of fillets and datapoints for each species.

The data acquisition setup included specialized hyperspectral imaging systems tailored for each spectral range. For the VNIR reflectance and fluorescence imaging, an in-house developed hyperspectral imaging system was employed [11]. A 150 W quartz tungsten lamp (Dolan Jenner, Boxborough, MA, USA) served as the light source for VNIR reflectance, while two UV narrowband light sources, each equipped with four 10 W, 365 nm LEDs (LED Engin, San Jose, CA, USA), were used for fluorescence imaging. The VNIR reflectance images were captured in 125 wavelengths ranging from 419 nm to 1007 nm, while the fluorescence images were acquired in 60 wavelengths ranging from 438 nm to 718 nm. The imaging system consisted of a 23 mm focal-length lens, an imaging spectrograph (Hyperspec-VNIR, Headwall Photonics), and a 14-bit electron-multiplying charge-coupled device (EMCCD) camera (Luca DL 604M, Andor Technology, South Windsor, CT, USA).

To capture reflectance images in the SWIR region, a separate hyperspectral imaging system was employed. This system utilized a custom-designed two-unit lighting system, with each unit containing four 150 W gold-coated halogen lamps with MR16 reflectors. The detection unit included a 25 mm focal-length lens and a hyperspectral camera equipped with a 16-bit mercury cadmium telluride array detector and an imaging spectrograph (Hyperspec-SWIR, Headwall Photonics, Fitchburg, MA, USA). The SWIR reflectance images were acquired in a wavelength range of 842 nm to 2532 nm, covering 287 wavelengths.

The push broom method was employed for image acquisition, utilizing a motorized linear translation stage to incrementally move the sample holder across the scanning line of the imaging spectrograph. The lens-to-sample distance was adjusted to ensure that the length of the instantaneous field of view (IFOV) slightly exceeded the length of the sample holder (150 mm), resulting in a spatial resolution of 0.4 mm/pixel along the length dimension. Sampling was performed along the width direction (100 mm) of the holder with a step size of 0.4 mm to match the spatial resolution of the length direction [11].

**Figure 2:**
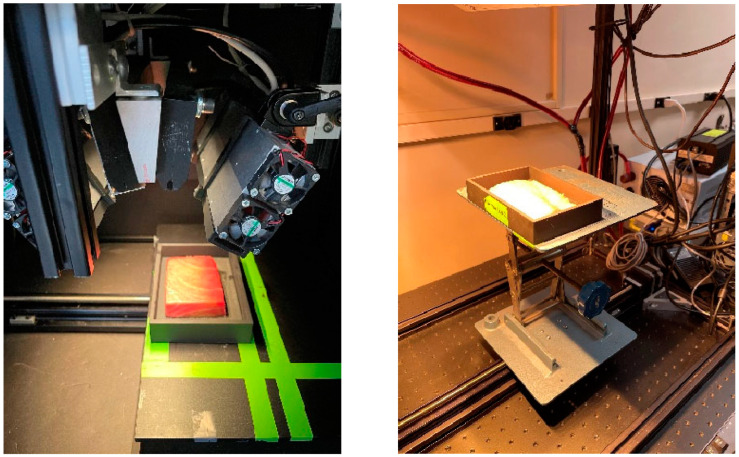
(**left**) Fluorescence and VNIR and (**right**) SWIR line-scan hyperspectral imaging systems. The VNIR reflectance images were captured from 419 nm to 1007 nm, while the fluorescence images were acquired from 438 nm to 718 nm. The SWIR reflectance images were taken from a wavelength range of 842 nm to 2532 nm.

The fish fillets were placed in customized sample holders created by a 3D printer using black thermoplastic. Each sample holder had an area of 150 × 100 mm^2^. Additional plates were printed to sit within the holder under the samples. Each sample rested on top of the plates within the holder to ensure consistent distance between samples and camera. Rust-Oleum black paint was applied to the sample holders and the plates to remove glare (Figure 2).

### 2.3. Preprocessing Methods

To ensure accurate data acquisition, several preprocessing steps were implemented. The background was masked and the original absolute intensities in CCD counts were converted to relative reflectance and fluorescence intensities by applying flat-field corrections to the VNIR and SWIR reflectance images, as well as the fluorescence images. Outlier detection was carried out by first creating spatial masks for each spectroscopic mode to separate the fish fillets from the background. The mean (μ) and standard deviation (σ) of the fish pixel intensities were then calculated over the entire fillet. Pixels in 10 × 10 regions were grouped together to make voxels, with the third-dimension denoting wavelengths with the aforementioned resolutions, mimicking independent fish fillet spectral-point measurements using the field of view of a fiber optic spectrometer in the development of our research group.

**Figure 3:**
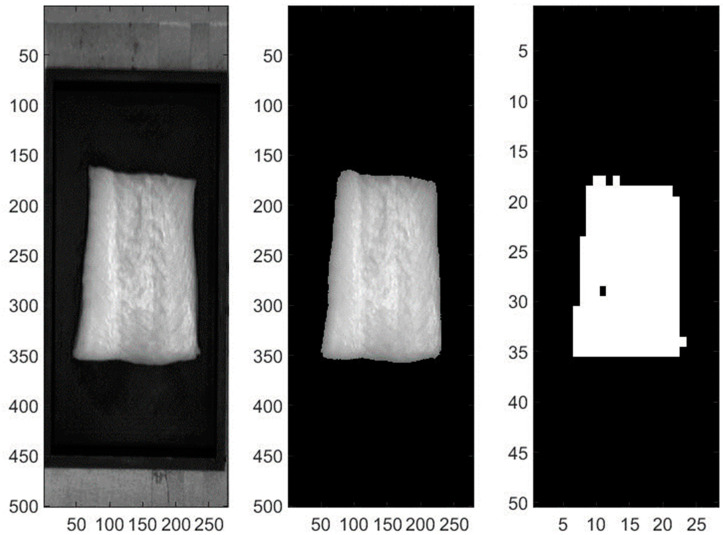
Data collection and processing are shown for a sablefish fillet. The raw VNIR image (**left**), with a mask applied to remove the background (**center**) and voxels of 10 × 10 pixels are formed with outliers removed (**right**), with the third-dimension denoting wavelengths, which are not displayed in this figure, as the images are taken from the 29th band (552 nm). Valid voxels are shown in white.

Voxels with more than 10% of pixels exceeding μ ± 2 σ were excluded to eliminate outliers. This voxel processing approach generated spatial masks for the VNIR and SWIR reflectance and fluorescence images [11]. Within these voxels, the spectra from the constituent pixels (100 pixels per voxel) would be averaged to output one spectrum per voxel to be used for analysis (Figure 3). This process was performed to simulate the point spectroscopy system.

The reflectance sample intensities were calibrated using white and dark references, which rendered all values between zero and one and allowed for consistency between different measurement periods. Equation (1) demonstrates the calibration process for the reflectance spectra. Additionally, the fluorescence spectra were adjusted by subtracting the dark, and then divided by a constant factor of 6000. This value was chosen as it was approximately the maximum spectral value found within the entirety of the data, other than one outlier sample.

(1) $$I_{sample,\;calibrated}=\frac{I_{sample}-I_{dark}}{I_{white}-I_{dark}}$$

**Figure 4:**
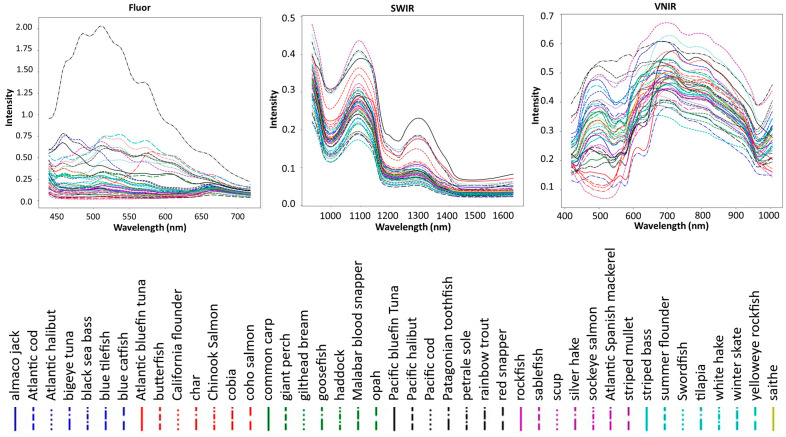
Averages of all spectra from each of the 43 fish species from (**left**) Fluor emission spectra for 365 nm excitation, (**middle**) VNIR and (**right**) SWIR.

Examples of spectra for each species and each spectroscopic mode can be seen below, in Figure 4.

### 2.4. Classification Methods

In this study, a Multilayer Perceptron (MLP) neural network was employed as the classification method for the global model. The MLP neural network is a feed-forward artificial neural network commonly used for supervised learning tasks, which aims to determine optimal weight values to create a nonlinear decision boundary while minimizing a defined cost function. The specific configuration of the MLP classifier utilized in this experiment consisted of two hidden layers. The first hidden layer contained 512 nodes, while the second hidden layer comprised 128 nodes. To introduce nonlinearity into the network, the rectified linear unit (ReLU) activation function was applied to the input and hidden layers. The ReLU activation function ensures that the output of each node is zero for negative inputs, and linearly increases for positive inputs, allowing the network to model complex relationships in the data. For the output layer, the softmax activation function was employed to generate the final classification decision. The softmax function normalizes the outputs of the network into a probability distribution, where each output represents the likelihood of the input belonging to a specific class. This allows for the prediction of the class label with the highest probability.

Two regularization techniques were then implemented to avoid overfitting. First, dropout regularization with a probability of 50% was applied to both hidden layers. Dropout randomly deactivates a proportion of the nodes during training, forcing the network to learn redundant representations and improve its generalization capabilities. Second, L2 kernel regularization, also known as weight decay, was employed on both hidden layers. This regularization technique involves adding a regularization term to the loss function that increases with the magnitude of the network’s weight vector. In this case, an L2 regularization factor (λ) of 0.0001 was utilized to prevent overfitting by discouraging large weight values. The cost function used for training the MLP classifier was defined as the complement of the multiclass classification accuracy, weighted by the number of samples per class. This cost function aimed to minimize misclassifications, while considering the imbalance in the dataset.

**Figure 5:**
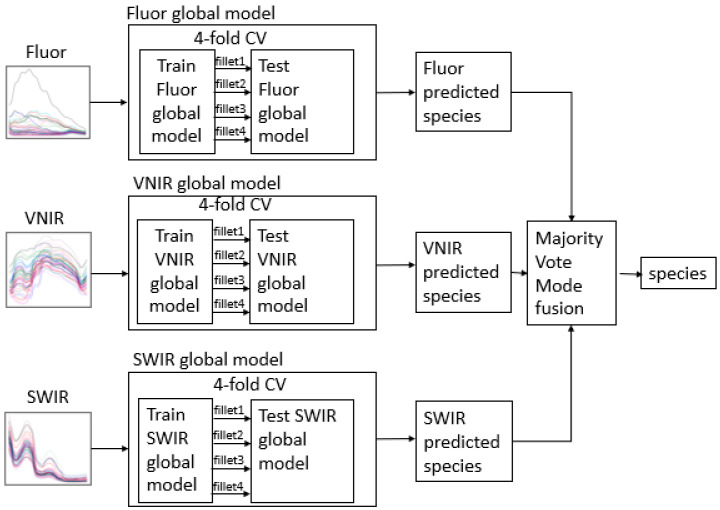
Global (single AI on all species) fusion model where one single ML model was trained on all species, before a majority vote between predictions from all three spectroscopic modes to obtain a final prediction; CV: Cross Validation.

For each spectroscopic mode, a 4-fold cross validation was performed, where a different fillet from among multiple fillets for each species was used exclusively as the test set of each fold. This ensured that not only does the model not “see” the test datapoint during training, but no other datapoints from the test fillet, either (Figure 5). 

### 2.5. Dispute Model Technique

**Figure 6:**
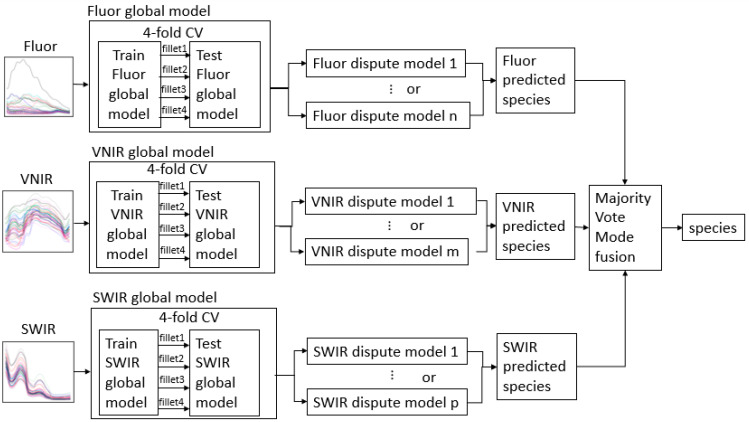
Hybrid fusion model where the global model was trained on all species, with the dispute models for each spectroscopic mode determining predictions for spectrally similar fish groupings. A final majority vote occurred to give a final prediction.

**Figure 7:**
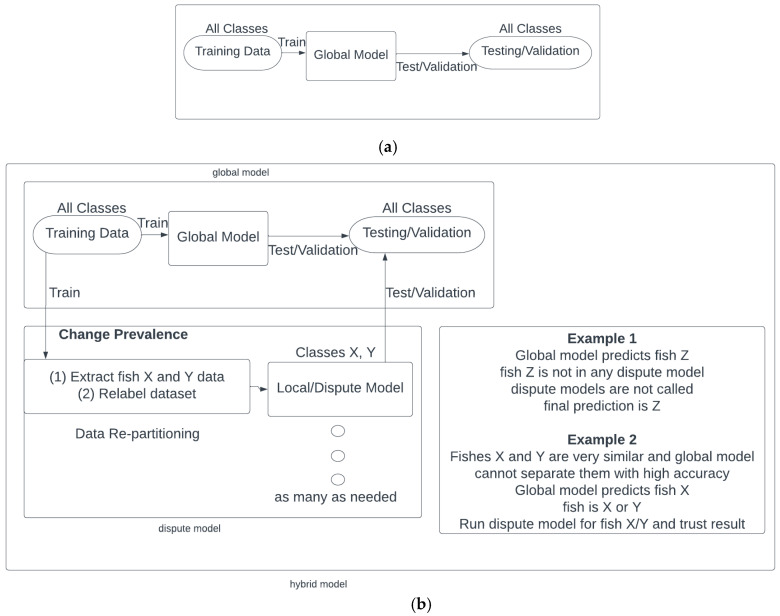
Schematic of (**a**) the global model and (**b**) hybrid (global integrated with dispute) model technique.

There are as many as 43 species/classes in our model. This high number of classes and similarities of spectra from groups of biologically close species make it difficult to achieve high levels of accuracy with a single model. Therefore, we devised a novel machine learning technique that improves the identification accuracies for all species by training specialized models that can differentiate between species of a similar underlying biology. Each of these local models learns to classify species of a group whose spectra are too close for one model to separate. These specialized models are called and run by the global model that was explained in the previous section, and are shown in Figure 6 and Figure 7. To determine how many specialized models were needed and the species in each of them, the test results of the global model were evaluated using the confusion matrix. When testing the global model, if the accuracy of a species was lower than a threshold, then that species became a member class of a specialized model, and is called a forming species. This specialized model was then trained on the forming species and the other species that the forming species was generally misclassified as. No species can be in more than one dispute model, because the members of the dispute groups must remain exclusive. Therefore, if one species is consistently being misclassified as multiple other species, a decision has to be made as to which of the low-performance species a dispute model will be formed with. Whichever combination of classes within the dispute models that attains the largest net positive change in performance will be chosen.

To train and evaluate the dispute models, the training data were relabeled to match each of the dispute models. Relabeled training data were fed into each dispute model to train via a one-dimensional convolutional neural network (1D CNN). The 1D CNN consisted of four layers in total, beginning with the input layer, which applied 64 filters of size five to the input data, with a stride value of four. The activation function used in the first layer was the scaled exponential linear unit (SELU).

The following three layers were dense (fully connected) layers. The second and third layers served as hidden layers, while the fourth layer acted as the output layer. The second and third dense layers had 128 units each, and the fourth dense layer had the number of classes in the given classification task, which was the number of species within each specific dispute model.

To extract features, the network used one-dimensional convolutional layers with 64 filters of size five in each hidden layer. The convolutional layers were followed by activation functions specific to each layer: the exponential linear unit (ELU) for the second layer, the Swish activation function for the third layer, and the softmax activation function for the output layer. Dropout regularization was applied after each dense layer, at a rate of 0.5. This helped to prevent overfitting by randomly setting a fraction of the inputs to 0 during training.

During training, the network optimized the learning rate with the Adam optimizer, an adaptive-learning-rate optimization algorithm. Categorical cross-entropy, commonly used for multi-class classification problems, was utilized as the loss function, as it measures the dissimilarity between the predicted class probabilities and the true class labels.

When given a measurement to classify, first the global model will predict a class. If the predicted class is not a forming species, then the global model’s predicted class will be deemed as the hybrid model’s prediction. However, if the global model’s prediction is among the forming species, the associated specialized model’s prediction will be the class predicted by the hybrid model (Figure 7). The dispute model technique proposed in this study is a supervised classification method and thus different from clustering methods.

### 2.6. Fusion of the Three Spectroscopic Modes within the Machine Learning Algorithm

To fuse the three different predictions from each spectroscopic mode, each of the three modes entered a majority voting system, where the species prediction with the most votes is output as the prediction of the global fusion model. Each spectroscopic mode was treated independently in developing its dispute model set. Therefore, each of the three spectroscopic modes has its own hybrid (global + dispute) model.

## 3. Results

### 3.1. Global Model Classification Results

**Figure 8:**
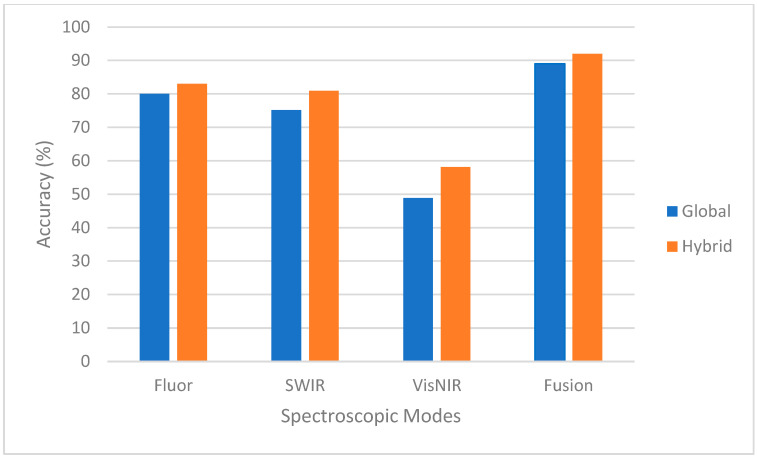
Accuracies for global and hybrid models for single spectroscopic modes and fusion.

Four-fold cross-validated classification accuracies for all species were obtained for each of the three single-mode spectroscopic modes using the global MLP models (Section 2.4). Accuracy/performance in this study is defined by the percentage of voxels of a species that are classified correctly. The predictions of the three modes then entered a majority vote system to determine the fused prediction (Section 2.6). The performance, averaged over all species, for single-mode fluorescence, VNIR, SWIR, and fusion of modes was 80%, 75.16%, 48.87%, and 89%, respectively (Figure 8). This demonstrates that the fusion of the three spectroscopic modes significantly improved the accuracies.

**Figure 9:**
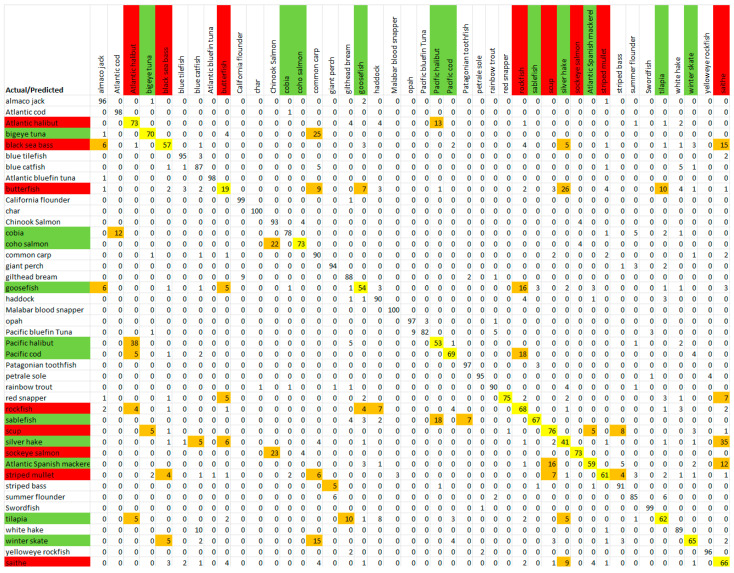
Confusion matrix of Fluor general model with accuracies in percentages. Yellow = low performance; green = dispute-model-forming species; red = low-performance species without a dispute model; orange = potential dispute-model members.

The confusion matrix of the datapoints for all species obtained from the Fluor mode global model is shown in Figure 9, as an example. This confusion matrix shows low-performance species (smaller accuracies on the diagonal of the confusion matrix) that are commonly misclassified, as well as the species that those low-performance species were incorrectly predicted as. This was the basis of the formation of dispute models. A Cohen’s kappa value was calculated for the confusion matrices, and is a statistic used to take into account chance agreement, which is not accounted for with the traditional percent agreement displayed within our confusion matrices [26]. A kappa value of 0.79 was calculated for the Fluor mode global model, which is categorized as substantial agreement, according to Cohen. A further kappa value was found for the results after the implementation of the dispute models, and will be discussed in the following section.

### 3.2. Results after the Formation and Implementation of Dispute Models

**Figure 10:**
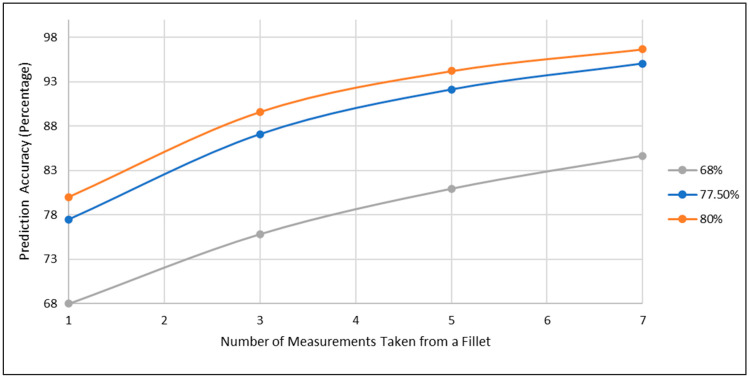
Improvement in species prediction accuracy for a fillet as the number of measurement points increases for fillets with 68%, 78% and 80% single-point measurement accuracy.

Subgroups were developed for the low-performance species found from the diagonal of the confusion matrices, and can be seen for the Fluor mode in Figure 9. Each subgroup consisted of one low-performance species, as well as the species for which the low-performance species was falsely predicted. When the accuracy for a specific species from the global fusion model is below a threshold, our dispute model technique will be applied to that species, in an attempt to improve its accuracy. A threshold performance of 78% has been chosen because with a baseline single measurement accuracy of 78% an overall fillet classification accuracy of 95% can be obtained with seven repeated measurements. With each measurement only taking seconds to complete, as many as seven measurements from a fillet can reasonably be completed without sacrificing rapidity. The improvement garnered from repeated measurements, based on a majority vote among an odd number of measurements from a fillet, is shown in Figure 10. For example, if three measurements are taken from a fillet, if two or three of the total three measurements predict the true species, then the majority vote makes the same prediction. This process can be extended to any odd number of measurements where a majority can be obtained, such as taking five or seven points. The total probability, *p_repeat_*, of the correct species being predicted by a majority vote between n measurements is given by Equation (2).

(2) $$p_{repeat}=\sum_{i=\left[\frac{n}{2}\right]+1}^{n}p^{i}{\left(1-p\right)}^{n-i}$$

where *p* is the probability of predicting the correct species from a single-point measurement and *n* is an odd number representing how many other points from that fillet have been sampled. As can be observed, if the accuracy of one correctly predicted point for a specific species is 78% or above, then seven or fewer measurements are enough to reach the accuracy of 95%.

**Figure 11:**
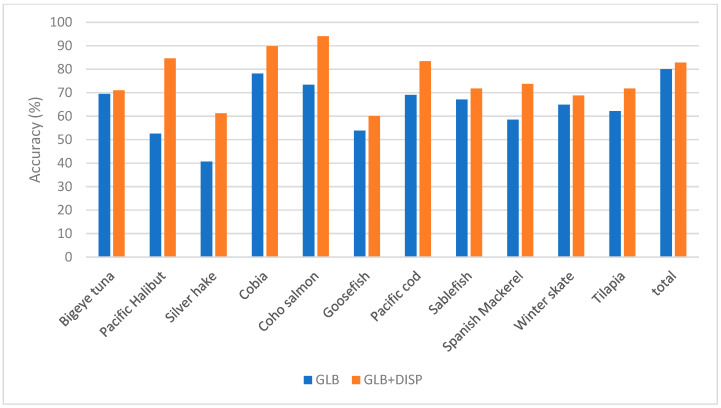
Improvement in the accuracy of fluorescence mode by applying dispute models on low-performing species.

**Figure 12:**
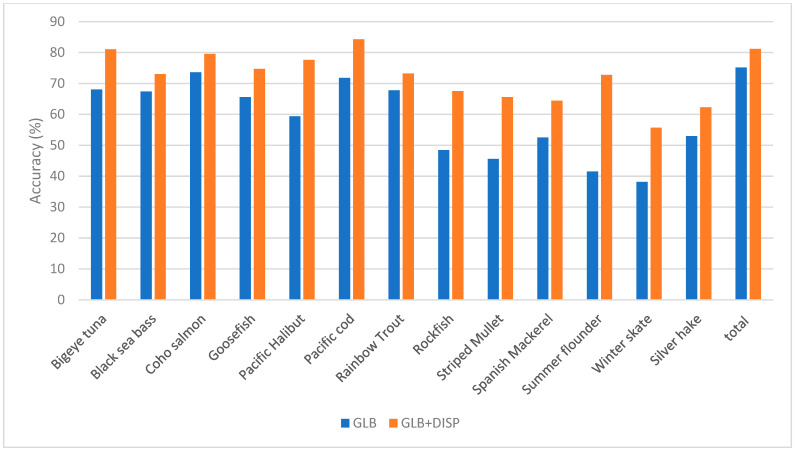
Improvement in the accuracy of VNIR mode by applying dispute models on low-performing species.

**Figure 13:**
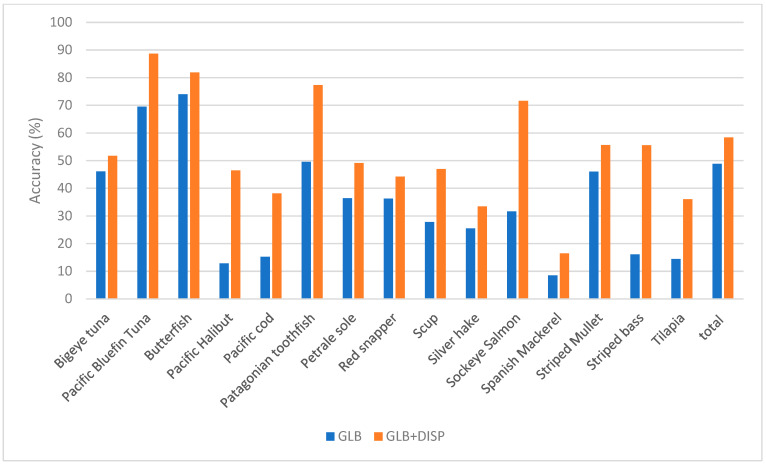
Improvement in the accuracy of SWIR mode by applying dispute models on low-performing species.

**Figure 14:**
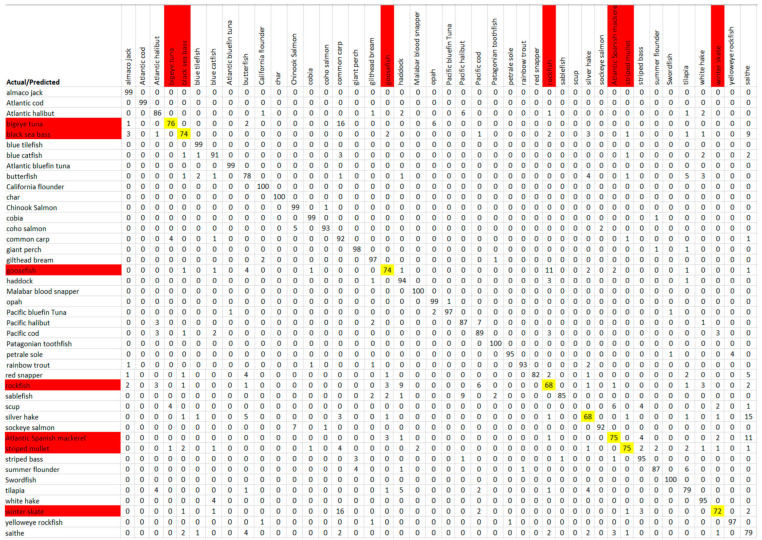
Confusion matrix with accuracies in percentages for fused hybrid model. Red = Low-performance species (the species that do not meet the 78% threshold, or 95% accuracy after seven measurements); Yellow = Low-performance accuracy (below the 78% threshold).

Table 4, Table 5 and Table 6, and Figure 11, Figure 12 and Figure 13 display which low-performance species were grouped with the corresponding commonly misclassified species, as well as the improvements in the accuracy for each subgroup. Figure 14 shows the confusion matrix for the fused hybrid model.

**Figure 15:**
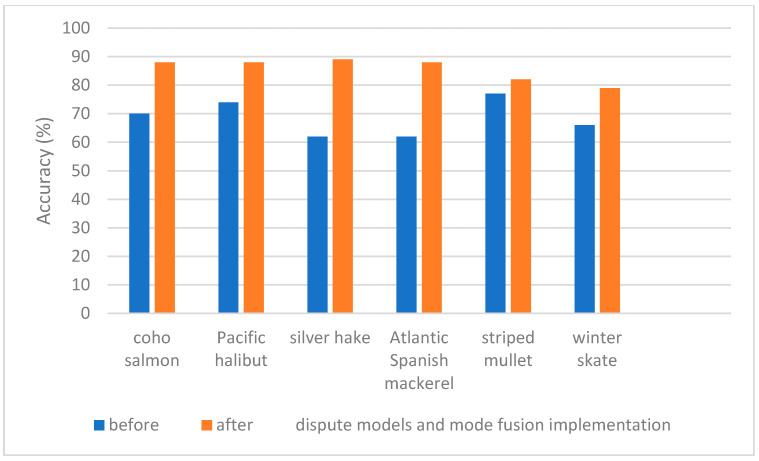
Accuracies before and after the implementation of dispute models and mode fusion.

The confusion matrix after the implementation of the dispute models and mode fusion is shown in Figure 14. The accuracies, averaged across species, for single modes as well as the fusion of modes are shown in Figure 15 and Table 7. The hybrid model performed well, yielding promising results, and just missed the 78% threshold where 95% accuracy can be obtained with seven rapid measurements in 7 of the 43 species. These lower-performing species are highlighted within Figure 14, and their results could be further improved with a larger dataset which incorporated more samples for each species. A Cohen’s kappa of 0.89 was calculated for the confusion matrix associated with the implementation of the dispute models and spectroscopic mode fusion (Figure 14). This value correlates to near-perfect agreement, and ensures that our performance observed within the confusion matrix did not occur by chance, but rather that our framework accurately classified the species and that the agreement observed is much beyond what would occur with the expected random chance of agreement [26].

## 4. Discussion

We measured Fluor, VNIR and SWIR spectra of 216 fish fillets from 43 different species. We then trained three MLP neural network models, one for each spectroscopic mode, and fused their decisions in a majority vote system. Simpler algorithms were also initially tested alongside the MLP neural network to decide which model should be implemented within our hierarchy of dispute models; LDA was the only model that was able to reach comparable results, while weighted k-nearest neighbors, support vector machine with a linear kernel, and Gaussian naïve Bayes achieved lower accuracies. MLP was chosen over LDA, however, as it displayed better performances when limiting the data to specific wavelengths, and as this framework will be implemented within a handheld system, the ability to still perform well with minimal data separated MLP from LDA as the better model for our purposes.

We then tested the MLP model against completely “unseen” fillets. The results displayed an average accuracy of approximately 89%. To improve performance, a novel approach based on a hierarchy of expert dispute models was developed. Dispute models specialize in differentiating similar fish species, and are called by the global model. This technique increased the average accuracies for Fluor, VNIR and SWIR spectroscopic modes by 3%, 6%, and 9%, respectively, and 3% for fused data. For some species this increase was as high as 40%. The hybrid model showed very promising results; however, there are eight species with accuracies lower than 95%, a performance level to be achieved if this technology is to compete with DNA testing, after seven measurements of a fillet. This shortcoming can be attributed to a lack of data, and will be overcome by a larger dataset in future studies. Also, because k-fold cross-validation can introduce bias and over-fitting, a future enlarged dataset can be used to improve generalization capabilities by setting data aside for external validation.

Other authors have also achieved high accuracies when classifying fish species by utilizing spectroscopy and machine learning approaches. Our approach, however, varies from all other research in differing aspects compared to each. While the studies produced by Chen and Ren [13,14] minced their fish, our approach can be conducted in real-time, on-site and with no extra preparation, as our results have been validated on fish fillets. Lv et al. identified fish species at a perfect rate with their methods, but their sample size only consisted of seven unique species. Simpler ML algorithms such as LDA that were used in their study have proven their efficacy on datasets with comparatively lower numbers of classes, but our dispute model framework provides a solution for the confusion that ensues when dealing with many classes/species, as is the case when dealing with many different fish within a market. Chen and Chauvin [14,15] both provided solutions with the ability to be applied to handheld systems, offering rapid and portable measurements as was carried out in our study, but again, their sample size was comparatively small, and does not classify as wide a range of species as seen within this research. De Graeve et al. [10] analyzed many samples and species, but this more comprehensive dataset does not have the means to be applied to a portable system that does not harm the samples in the process. The ML framework and proposed system demonstrated within this manuscript is the only current approach with the ability to identify a large range of different fish species accurately, rapidly, non-destructively, and without any skilled labor. Further research should be conducted to examine the ability of the method to identify “unseen” fillets collected at different time points from those in the database, due to natural variability in the properties of fish fillets.

This study demonstrates how multi-mode spectroscopy and machine learning can rapidly, accurately, non-destructively, and with low cost, identify fish species. Earlier work carried out by our same team had also demonstrated how accurate determination of fish freshness can be achieved through the same system in tandem with machine learning techniques [16]. This prior research into freshness classification displayed how the SWIR mode achieved the best results when classifying fish freshness, while within this manuscript, Fluor was found to be the highest-performing spectroscopic mode for identifying fish species. The combination of these experiments further proves the merits of a system that can provide multiple modes of spectroscopy with fusion AI to provide a comprehensive solution for identifying both the species and the freshness of fish fillets.

Our group is developing a handheld multi-mode point spectroscopy system for food quality, authentication and traceability (QAT). The fish species identification study presented in this work, and our fish freshness assessment [16], which use hyperspectral imaging systems, are feasibility studies for proving the performance and accuracy of our QAT technology. Our QAT device has been used at Chapman University on many fish species, and preliminary results show high accuracies. This technology can be expanded to many other food and agricultural products. The dispute model framework is also not limited to only this application, but can be extended to additional applications outside of fish species identification.

To enhance the capabilities of seafood supply-chain systems, we are exploring the incorporation of transformative technologies such as blockchain. Specifically, we aim to integrate blockchain technology with Internet of Things (IoT)-enabled devices like our handheld spectroscopic devices. Blockchain’s resilient features, including immutability, decentralization, verifiability, and trust, complemented by AI technology’s intelligent capabilities, can significantly reduce food fraud. By assessing fish freshness, estimating shelf life, and preventing food adulteration and related risks, this approach will contribute to strengthening food supply-chain systems and ensuring consumer safety.

## 5. Patents

System and method for assessing product; Application; Publication/Patent Number: US20230142722A1; Publication Date: 5 November 2023; Application Number: US17/980,996; Filing Date: 11 April 2022; Inventor: Vasefi, Fartash; Barton, Kenneth Edward; Bearman, Gregory; Kashani Zadeh, Hossein; Akhbardeh, Alireza; Assignee: SafetySpect Inc.; IPC: G01N33/12.

## Figures and Tables

**Table 1:** Health risks due to mislabeled fish based on actual incidents of species substitution [4].

Actual Market Name	Mislabeled as	Hazards
escolar	sea bass, white tuna	gempylotoxin, histamine
puffer fish	monkfish	tetrodotoxin, paralytic shellfish poisoning
Atlantic Spanish mackerel	kingfish	parasites, histamine, ciguatera fish poisoning
basa	grouper	environmental chemical contaminants and pesticides
grouper	cod	parasites, ciguatera fish poisoning

**Table 2:** Comparing fish fillet species identification methods; sal = salary; equip = equipment; Colors green, orange and red represent from the most to the least desirable.

Method	Prep	Duration	Cost	In Situ	Skill	Destructive
**Visual inspection**	no	min	sal	yes	yes	no
**DNA Barcoding**	yes	d	lab supplies, equip, sal	no	yes	yes
**Real-time PCR**	yes	min-h	lab supplies, equip, sal	yes	yes	yes
**Spectroscopy**	no	s	device	yes	no	no

**Table 3:** The species and the number of fillets and datapoints; smp = number of fillets; points = number of datapoints.

Common Name	Scientific Name	Smp	Points	Common Name	Scientific Name	Smp	Points
almaco jack	*Seriola rivoliana*	4	1157	Pacific bluefin tuna	*Thunnus orientalis*	4	918
Atlantic bluefin tuna	*Thunnus thynnus*	6	1231	Pacific cod	*Gadus macrocephalus*	4	1619
Atlantic cod	*Gadus morhua*	4	1322	Pacific halibut	*Hippoglossus stenolepis*	5	1769
Atlantic halibut	*Hippoglossus hippoglossus*	5	1519	Patagonian toothfish	*Dissostichus eleginoides*	8	1437
Atlantic Spanish mackerel	*Scomberomorus maculatus*	4	1563	petrale sole	*Eopsetta jordani*	6	2253
bigeye tuna	*Thunnus obesus*	8	1086	rainbow trout	*Oncorhynchus mykiss*	9	3289
black sea bass	*Centropristis striata*	5	1529	red snapper	*Lutjanus campechanus*	8	4017
blue catfish	*Ictalurus furcatus*	4	1450	rockfish	*Sebastes norvegicus*	12	3197
blue tilefish	*Lopholatilus chamaeleonticeps*	4	2334	sablefish	*Anoplopoma fimbria*	6	954
butterfish	*Peprilus triacanthus*	4	726	saithe	*Pollachius virens*	4	2251
California flounder	*Paralichthys californicus*	4	1016	scup	*Stenotomus chrysops*	5	1090
char	*Salvelinus alpinu*	4	1165	silver hake	*Merluccius bilinearis*	4	1791
Chinook salmon	*Oncorhynchus tshawytscha*	5	1630	sockeye salmon	*Oncorhynchus nerka*	4	1033
cobia	*Rachycentron canadum*	4	1235	striped bass	*Morone saxatilis*	5	1552
coho salmon	*Oncorhynchus kisutch*	5	894	striped mullet	*Mugil cephalus*	4	1730
common carp	*Cyprinus carpio*	4	2014	summer flounder	*Paralichthys dentatus*	5	1521
giant perch	*Lates calcarifer*	4	1046	swordfish	*Xiphias gladius*	4	789
gilthead bream	*Sparus aurata*	6	1314	tilapia	*Oreochromis sp*	6	1477
goosefish	*Lophius americanus*	4	1304	white hake	*Urophycis tenuis*	5	1631
haddock	*Melanogrammus aeglefinus*	4	1193	winter skate	*Leucoraja ocellata*	4	1839
Malabar blood snapper	*Lutjanus malabaricus*	4	5530	yelloweye rockfish	*Sebastes ruberrimus*	4	1197
opah	*Lampris guttatus*	4	913				

**Table 4:** Fluorescence dispute models and accuracies for fused global and fused hybrid models.

Fluor	Species			Glb (%)	Glb + Dispt (%)	Δ (%)
**Subgroup**	Target	Added to Submodel				
**1**	silver hake	saithe	blue catfish	40.65	61.19	20.54
**2**	Pacific halibut	Atlantic halibut		52.59	84.58	31.99
**3**	bigeye tuna	common carp		69.51	70.97	1.46
**4**	cobia	Atlantic cod		78.12	89.83	11.71
**5**	coho salmon	chinook Salmon		73.39	94.02	20.63
**6**	goosefish	almaco jack	butterfish	53.83	60.01	6.18
**7**	Pacific cod	rockfish		69.07	83.46	14.39
**8**	sablefish	Patagonian toothfish		67.08	71.75	4.67
**9**	Atlantic Spanish mackerel	scup		58.53	73.74	15.21
**10**	winter skate	black sea bass		64.9	68.82	3.92
**11**	tilapia	gilthead bream		62.16	71.81	9.65
**Total**				80	82.82	2.82

**Table 5:** VNIR dispute models and accuracies for fused global and fused hybrid models.

VNIR	Species			Glb (%)	Glb + Dispt (%)	Δ (%)
Subgroup	Target	Added to Submodel				
**1**	bigeye tuna	Malabar blood snapper	opah	68.06	81.03	12.97
**2**	black sea bass	white hake		67.36	72.99	5.63
**3**	coho salmon	chinook salmon		73.59	79.57	5.98
**4**	goosefish	common carp		65.56	74.69	9.13
**5**	Pacific halibut	yelloweye rockfish	Atlantic halibut	59.34	77.61	18.27
**6**	Pacific cod	haddock	Patagonian toothfish	71.77	84.25	12.48
**7**	rainbow trout	char		67.77	73.21	5.44
**8**	rockfish	Atlantic cod	giant perch	48.4	67.53	19.13
**9**	striped mullet	scup	almaco jack	45.57	65.55	19.98
**10**	Atlantic Spanish mackerel	red snapper		52.53	64.43	11.9
**11**	summer flounder	California flounder	tilapia	41.49	72.78	31.29
**12**	winter skate	blue catfish		38.12	55.68	17.56
**13**	silver hake	petrale sole	saithe	52.93	62.26	9.33
**Total**				75.16	81.14	5.98

**Table 6:** SWIR dispute models and accuracies for fused global and fused hybrid models.

SWIR	Species				Glb (%)	Glb + Dispt (%)	Δ (%)
Subgroup	Target	Added to Submodel					
**1**	bigeye tuna	opah			46.08	51.72	5.64
**2**	Pacific bluefin tuna	Atlantic bluefin tuna			69.53	88.65	19.12
**3**	butterfish	swordfish			74.02	81.87	7.85
**4**	Pacific halibut	blue tilefish	Malabar blood snapper	saithe	12.82	46.45	33.63
**5**	Pacific cod	Atlantic cod			15.24	38.14	22.9
**6**	Patagonian toothfish	sablefish	char	chinook Salmon	49.59	77.3	27.71
**7**	petrale sole	cobia			36.38	49.15	12.77
**8**	red snapper	white hake			36.25	44.2	7.95
**9**	scup	rainbow trout	common carp		27.81	46.94	19.13
**10**	silver hake	winter skate			25.45	33.41	7.96
**11**	sockeye salmon	coho salmon	goosefish		31.63	71.59	39.96
**12**	Atlantic Spanish mackerel	black sea bass			8.47	16.43	7.96
**13**	striped mullet	giant perch			46.04	55.61	9.57
**14**	striped bass	blue catfish			16.06	55.57	39.51
**15**	tilapia	Atlantic halibut	summer flounder		14.44	36.02	21.58
**Total**					48.87	58.35	9.48

**Table 7:** Single and fusion-of-mode accuracies before and after the implementation of dispute models.

Mode	Accuracies		Δ Accuracies
	Global (%)	Hybrid (%)	Hybrid-Global (%)
**Fluorescence**	80	83	3
**VNIR**	75	81	6
**SWIR**	49	58	9
**Fusion**	89	92	3

## Data Availability

Data available on request, due to privacy restrictions.
